# An emerging consensus for open evaluation: 18 visions for the future of scientific publishing

**DOI:** 10.3389/fncom.2012.00094

**Published:** 2012-11-15

**Authors:** Nikolaus Kriegeskorte, Alexander Walther, Diana Deca

**Affiliations:** ^1^Medical Research Council Cognition and Brain Sciences UnitCambridge, UK; ^2^Institute of Neuroscience, Technische Universität MünchenMunich, Germany

A scientific publication system needs to provide two basic services: access and evaluation. The traditional publication system restricts the access to papers by requiring payment, and it restricts the evaluation of papers by relying on just 2–4 pre-publication peer reviews and by keeping the reviews secret. As a result, the current system suffers from a lack of quality and transparency of the peer review process, and the only immediately available indication of a new paper's quality is the prestige of the journal it appeared in.

Open access (OA) is now widely accepted as desirable and is beginning to become a reality. However, the second essential element, evaluation, has received less attention. Open evaluation (OE), an ongoing post-publication process of transparent peer review and rating of papers, promises to address the problems of the current system and bring scientific publishing into the twenty-first century.

Evaluation steers the attention of the scientific community, and thus the very course of science. For better or worse, the most visible papers determine the direction of each field, and guide funding and public policy decisions. Evaluation, therefore, is at the heart of the entire endeavor of science. As the number of scientific publications explodes, evaluation, and selection will only gain importance. A grand challenge of our time, therefore, is to design the future system, by which we evaluate papers and decide which ones deserve broad attention and deep reading. However, it is unclear how exactly OE and the future system for scientific publishing should work. This motivated us to edit the Research Topic “Beyond open access: visions for open evaluation of scientific papers by post-publication peer review” in Frontiers in Computational Neuroscience. The Research Topic includes 18 papers, each going beyond mere criticism of the status quo and laying out a detailed vision for the ideal future system. The authors are from a wide variety of disciplines, including neuroscience, psychology, computer science, artificial intelligence, medicine, molecular biology, chemistry, and economics.

The proposals could easily have turned out to contradict each other, with some authors favoring solutions that others advise against. However, our contributors' visions are largely compatible. While each paper elaborates on particular challenges, the solutions proposed have much overlap, and where distinct solutions are proposed, these are generally compatible. This puts us in a position to present our synopsis here as a coherent blueprint for the future system that reflects the consensus among the contributors.^1^ Each section heading below refers to a design feature of the future system that was a prevalent theme in the collection. If the feature was overwhelmingly endorsed, the section heading below is phrased as a statement. If at least two papers strongly advised against the feature, the section heading is phrased as a question. Figure 1 visualizes to what extent each paper encourages or discourages the inclusion of each design feature in the future system. The ratings used in Figure 1 have been agreed upon with the authors of the original papers.^2^

**Figure 1:**
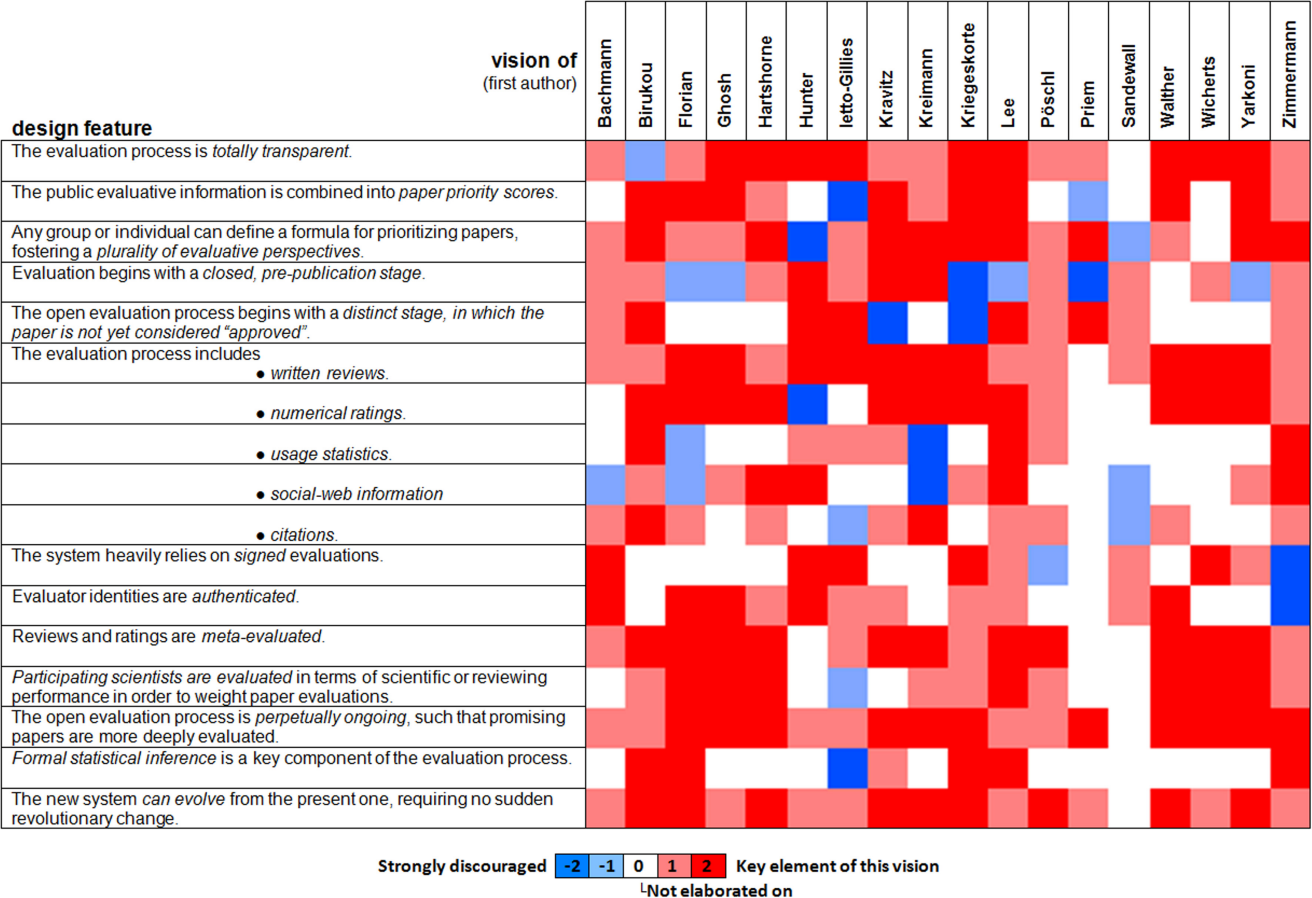
**Overview of key design features across the 18 visions**. The design features on the left capture major recurrent themes that were addressed (positively or negatively) in the Research Topic on OE. The columns indicate to what extent each design feature is a key element (red), actively endorsed (light red), not elaborated upon (white), discouraged (light blue), or strongly discouraged (blue) in each of the 18 visions. Overall, there is wide agreement on the usefulness of most of the features (prevalence of light red and red) and limited controversy (red and blue cells in the same row), indicating an emerging consensus. The 18 visions are indicated by their first author in alphabetical order at the top. The papers are Bachmann (2011); Birukou et al. (2011); Florian (2012); Ghosh et al. (2012); Hartshorne and Schachner (2012); Hunter (2012); Ietto-Gillies (2012); Kravitz and Baker (2011); Kreiman and Maunsell (2011); Kriegeskorte (2012); Lee (2012); Pöschl (2012); Priem and Hemminger (2012); Sandewall (2012); Walther and van den Bosch (2012); Wicherts et al. (2012); Yarkoni (2012), and Zimmermann et al. (2012).

## Synopsis of the emerging consensus

### The evaluation process is totally transparent

Almost all of the 18 visions favor *total transparency*. Total transparency means that all reviews and ratings are instantly published. This is in contrast to current practice, where the community is excluded and reviews are initially only visible to editors and later on to the authors (and ratings are often only visible to editors). Such secrecy opens the door to self-serving reviewer behavior, especially when the judgments are inherently subjective, such as the judgment of the overall significance of a paper. In a secret reviewing system, the question of a paper's significance may translate in some reviewers' minds to the question “How comfortable am I with this paper gaining high visibility now?” In a transparent evaluation system, the reviews and reviewers are subject to public scrutiny, and reviewers are thus more likely to ask themselves the more appropriate question “How likely is it that this paper will ultimately turn out to be important?”

### The public evaluative information is combined into paper priority scores

In a totally transparent evaluation process, the evaluative information (including reviews and ratings) is publicly available. Most of the authors suggest the use of functions that combine the evaluative evidence into an overall *paper priority score* that produces a ranking of all papers. Such a score could be computed as an average of the ratings. The individual ratings could be weighted in the average, so as to control the relative influence of different rating scales (e.g., reliability vs. novelty vs. importance of the claims) and to give greater weight to raters that are either highly regarded in the field (by some quantitative measure, such as the h-index) or have proved to be reliable raters in the past.

### Any group or individual can define a formula for prioritizing papers, fostering a plurality of evaluative perspectives

Most authors support the idea that a *plurality of evaluative perspectives* on the literature is desirable. Rather than creating a centralized black-box system that ranks the entire literature, any group or individual should be enabled to access the evaluative information and combine it by an arbitrary formula to prioritize the literature. A constant evolution of competing priority scores will also make it harder to manipulate the perceived importance of a paper.

### Should evaluation begin with a closed, pre-publication stage?

Whether a *closed, pre-publication stage* of evaluation (such as the current system's secret peer review) is desirable is controversial. On the one hand, the absence of any pre-publication filtering may open the gates to a flood of low-quality publications. On the other hand, providing permanent public access to a wide range of papers, including those that do not initially meet enthusiasm, may be a strength rather than a weakness. Much brilliant science was initially misunderstood. Pre-publication filtering comes at the cost of a permanent loss of value through errors in the initial evaluations. The benefit of publishing all papers may, thus, outweigh the cost of providing the necessary storage and access. “Publish, then filter” is one of the central principles that lend the web its power (Shirky, 2008). It might work equally well in science as it does in other domains, with *post-publication* filtering preventing the flood from cluttering our view of the literature.

### Should the open evaluation begin with a distinct stage, in which the paper is not yet considered “approved”?

Instead of a closed, pre-publication evaluation, we could define a *distinct initial stage of the post-publication open evaluation* that determines whether a paper receives an “approved” label. Whether this is desirable is controversial among the 18 visions. One argument in favor of an “approved” label is that it could serve the function of the current notion of “peer reviewed science,” suggesting that the claims made are somewhat reliable. However, the strength of post-publication OE is ongoing and continuous evaluation. An “approved” label would create an artificial dichotomy based on an arbitrary threshold (on some paper evaluation function). It might make it more difficult for the system to correct its errors as more evaluative evidence comes in (unless papers can cross back over to the “unapproved” state). Another argument in favor of an initial distinct stage of OE is that it could serve to incorporate an early round of review and revision. The authors could choose to either accept the initial evaluation, or revise the paper and trigger re-evaluation. However, revision and re-evaluation would be possible at any point of an open evaluation process anyway. Moreover, authors can always seek informal feedback (either privately among trusted associates or publicly via blogs) prior to formal publication.

### The evaluation process includes written reviews, numerical ratings, usage statistics, social-web information, and citations

There is a strong consensus that the OE process should include *written reviews* and *numerical ratings*. These classical elements of peer review continue to be useful. They represent explicit expert judgments and serve an important function that is distinct from the function of *usage statistics* and *social-web information*, which are also seen as useful by some of the authors. In contrast to explicit expert judgments, usage statistics, and social-web information may highlight anything that receives attention (of the positive or negative variety), thus potentially valuing buzz and controversy over high-quality science. Finally, *citations* provide a slow signal of paper quality, emerging years after publication. Because citations are slow to emerge, they cannot replace the other signals. However, they arguably provide the ultimately definitive signal of a paper's de-facto importance.

### The system utilizes signed (along with unsigned) evaluations

*Signed evaluations* are a key element of five of the visions, only one vision strongly discourages heavy reliance on signed evaluations. When an evaluation is signed, it affects the evaluator's reputation. High-quality signed evaluations can help build a scientist's reputation (thus motivating scientists to contribute). Conversely, low-quality signed evaluations can hurt a scientist's reputation (thus motivating high standards in rating and reviewing). Signing creates an incentive for objectivity and a disincentive for self-serving judgments. But as signing adds weight to the act of evaluation, it might also create hesitation. Hesitation to provide a rash judgment may be desirable, but the system does require sufficient participation. Moreover, signing may create a disincentive to present critical arguments as evaluators may fear potential social consequences of their criticism. The OE system should therefore collect both signed and unsigned evaluations, and combine the advantages of these two types of evaluation.

### Evaluators' identities are authenticated

*Authentication of evaluator identities* is a key element of five of the visions, one vision strongly discourages it. Authentication could be achieved by requiring login with a password before submitting evaluations. Authenticating the evaluator's identity does not mean that the evaluator has to publicly sign the evaluation, but would enable the system to exclude lay people from the evaluation process and to relate multiple reviews and ratings provided by the same person. This could be useful for assessing biases and estimating the predictive power of the evaluations. Arguments against authenticating evaluator identities (unless the evaluator chooses to sign) are that it creates a barrier to participation and compromises transparency (the “system,” but not the public knows the identity). However, authentication could use public aliases, allowing virtual evaluator identities (similar to blogger identities) to be tracked without any secret identity tracking. Note that (1) anonymous, (2) authenticated-unsigned, and (3) authenticated-signed evaluations each have different strengths and weaknesses and could all be collected in the same system. It would then fall to the designers of paper evaluation functions to decide how to optimally combine the different qualities of evaluative evidence.

### Reviews and ratings are meta-evaluated

Most authors suggest *meta-evaluation of individual evaluations*. One model for meta-evaluation is to treat reviews and ratings like papers, such that paper evaluations and meta-evaluations can utilize the same system. Paper evaluation functions could retrieve meta-evaluations recursively and use this information for weighting the primary evaluations of each paper. None of the contributors to the Research Topic object to meta-evaluation.

### Participating scientists are evaluated in terms of scientific or reviewing performance in order to weight paper evaluations

Almost all authors suggest that the system *evaluate the evaluators*. Evaluations of evaluators would be useful for weighting the multiple evaluations a given new paper receives. Note that this will require some form of authentication of the evaluators' identities. Scientists could be evaluated by combining the evaluations of their publications. A citation-based example of this is the h-index, but the more rapidly available paper evaluations provided by the new system could also be used to evaluate an individual's scientific performance. Moreover, the predictive power of a scientist's previous evaluations could be estimated as an index of reviewing performance. An evaluation might be considered predictive to the extent that it deviates from previous evaluations, but matches later aggregate opinion.

### The open evaluation process is perpetually ongoing, such that promising papers are more deeply evaluated

Almost all authors suggest a *perpetually ongoing OE* process. Ongoing evaluation means that there is no time limit on the evaluation process for a given paper. This enables the OE process to accumulate deeper and broader evaluative evidence for promising papers, and to self-correct when necessary, even if the error is only discovered long after publication. Initially exciting papers that turn out to be incorrect could be debunked. Conversely, initially misunderstood papers could receive their due respect when the field comes to appreciate their contribution. None of the authors objects to perpetually ongoing evaluation.

### Formal statistical inference is a key component of the evaluation process

Many of the authors suggest a role for *formal statistical inference in the evaluation process*. Confidence intervals on evaluations would improve the way we allocate our attention, preventing us from preferring papers that are not significantly preferable and enabling us to appreciate the full range of excellent contributions, rather than only those that find their way onto a stage of limited size, such as the pages of Science and Nature. To the extent that excellent papers do not significantly differ in their evaluations, the necessary selection would rely on content relevance.

### The new system can evolve from the present one, requiring no sudden revolutionary change

Almost all authors suggest that *the ideal system for scientific publishing can evolve* from the present one, requiring no sudden revolutionary change. The key missing element is a powerful general OE system. An OE system could initially serve to more broadly and deeply evaluate papers published in the current system. Once OE has proven its power and its evaluations are widely trusted, traditional pre-publication peer review will no longer be needed to establish a paper as part of the literature. Although the ideal system can evolve, it might take a major public investment (comparable to the establishment of PubMed) to provide a truly transparent, widely trusted OE system that is independent of the for-profit publishing industry.

## Concluding remarks

OA and OE are the two complementary elements that will bring scientific publishing into the twenty-first century. So far scientists have left the design of the evaluation process to journals and publishing companies. However, the steering mechanism of science should be designed by scientists. The cognitive, computational, and brain sciences are best prepared to take on this task, which will involve social and psychological considerations, software design, modeling of the network of scientific papers and their interrelationships, and inference on the reliability and importance of scientific claims. Ideally, the future system will derive its authority from a scientific literature on OE and on methods for inference from the public evaluative evidence. We hope that the largely converging and compatible arguments in the papers of the present collection will provide a starting point.

## Footnotes

^1^The consensus, of course, is only among the contributors to this collection. A consensus among the scientific community at large has yet to be established. Note that scientists critical of the general idea of OE would not have chosen to contribute here. Nevertheless, assuming OE is seen as desirable, the collection does suggest that independent minds will produce compatible visions for how to implement it.

^2^With the exception of Erik Sandewall, whom we could not reach before this piece went to press.

## References

[B1] Bachmann T. (2011). Fair and open evaluation may call for temporarily hidden authorship, caution when counting the votes, and transparency of the full pre-publication procedure. Front. Comput. Neurosci. 5:61 10.3389/fncom.2011.00061 22232590 PMC3247677

[B2] Birukou A. Wakeling J. R. Bartolini C. Casati F. Marchese M. Mirylenka K. (2011). Alternatives to peer review: novel approaches for research evaluation. Front. Comput. Neurosci. 5:56 10.3389/fncom.2011.00056 22174702 PMC3237011

[B3] Florian R. V. (2012). Aggregating post-publication peer reviews and ratings. Front. Comput. Neurosci. 6:31 10.3389/fncom.2012.00031 22661941 PMC3357530

[B4] Ghosh S. S. Klein A. Avants B. Millman K. J. (2012). Learning from open source software projects to improve scientific review. Front. Comput. Neurosci. 6:18 10.3389/fncom.2012.00018 22529798 PMC3328792

[B5] Hartshorne J. K. Schachner A. (2012). Tracking replicability as a method of post-publication open evaluation. Front. Comput. Neurosci. 6:8 10.3389/fncom.2012.00008 22403538 PMC3293145

[B6] Hunter J. (2012). Post-publication peer review: opening up scientific conversation. Front. Comput. Neurosci. 6:63 10.3389/fncom.2012.00063 22969719 PMC3431010

[B7] Ietto-Gillies G. (2012). The evaluation of research papers in the XXI century. The Open Peer Discussion system of the World Economics Association. Front. Comput. Neurosci. 6:54 10.3389/fncom.2012.00054 22891057 PMC3413091

[B8] Kravitz D. J. Baker C. I. (2011). Toward a new model of scientific publishing: discussion and a proposal. Front. Comput. Neurosci. 5:55 10.3389/fncom.2011.00055 22164143 PMC3230039

[B9] Kreiman G. Maunsell J. (2011). Nine criteria for a measure of scientific output. Front. Comput. Neurosci. 5:48 10.3389/fncom.2011.00048 22102840 PMC3214728

[B10] Kriegeskorte N. (2012). Open evaluation: a vision for entirely transparent post-publication peer review and rating for science. Front. Comput. Neurosci. 6:79 10.3389/fncom.2012.00079 23087639 PMC3473231

[B11] Lee C. (2012). Open peer review by a selected-papers network. Front. Comput. Neurosci. 6:1 10.3389/fncom.2012.00001 22291635 PMC3264905

[B12] Pöschl U. (2012). Multi-stage open peer review: scientific evaluation integrating the strengths of traditional peer review with the virtues of transparency and self-regulation. Front. Comput. Neurosci. 6:33 10.3389/fncom.2012.00033 22783183 PMC3389610

[B13] Priem J. Hemminger B. M. (2012). Decoupling the scholarly journal. Front. Comput. Neurosci. 6:19 10.3389/fncom.2012.00019 22493574 PMC3319915

[B14] Sandewall E. (2012). Maintaining live discussion in two-stage open peer review. Front. Comput. Neurosci. 6:9 10.3389/fncom.2012.00009 22363282 PMC3282940

[B15] Shirky C. (2008). Here Comes Everybody: The Power of Organizing Without Organizations. New York, NY: Penguin Press

[B16] Walther A. van den Bosch J. F. (2012). FOSE: a framework for open science evaluation. Front. Comput. Neurosci. 6:32 10.3389/fncom.2012.00032 22754522 PMC3385586

[B17] Wicherts J. M. Kievit R. A. Bakker M. Borsboom D. (2012). Letting the daylight in: Reviewing the reviewers and other ways to maximize transparency in science. Front. Comput. Neurosci. 6:20 10.3389/fncom.2012.00020 22536180 PMC3332228

[B18] Yarkoni T. (2012). Designing next-generation platforms for evaluating scientific output: what scientists can learn from the social web. Front. Comput. Neurosci. 6:72 10.3389/fncom.2012.00072 23060783 PMC3461500

[B19] Zimmermann J. Roebroeck A. Uludag K. Sack A. T. Formisano E. Jansma B. (2012). Network-based statistics for a community driven transparent publication process. Front. Comput. Neurosci. 6:11 10.3389/fncom.2012.00011 22403537 PMC3293411

